# Laparoscopy treatment of liver abscess secondary to an unusual foreign body (rosemary twig)

**DOI:** 10.4322/acr.2021.317

**Published:** 2021-08-20

**Authors:** André Thá Nassif, Victor Hugo Granella, Tulio Rucinski, Bruno Landal Cavassin, Alesandra Bassani, Lucas Thá Nassif

**Affiliations:** 1 Hospital Santa Casa de Curitiba, Department of General Surgery, Curitiba, PR, Brasil; 2 Pontifícia Universidade Católica do Paraná, School of Medicine, Curitiba, PR, Brasil

**Keywords:** Liver Abscess, Foreign-Body Migration, Laparoscopy, General Surgery

## Abstract

A hepatic abscess caused by a swallowed foreign body is a rare and challenging diagnosis. Most patients have nonspecific symptoms, and more than 90% of patients do not remember having swallowed it, which occurred accidentally. In this setting, fish bones, chicken bones, and toothpicks are the most found foreign bodies. We reported the case of a 54-year-old male patient admitted with abdominal pain and intermittent fever. He was diagnosed with liver abscess and treated successfully with antibiotics and a laparoscopic procedure; a rosemary twig was found during the abscess drainage procedure. Furthermore, a literature review of 22 cases of laparoscopic treated liver abscesses associated with a foreign body was made.

## INTRODUCTION

The ingestion of foreign bodies is not an uncommon incident; however, complications are rare and correspond to less than 1% of cases.^1^^,^ 2 The formation of a liver abscess, after perforation of the gastrointestinal tract, caused by the ingestion of a foreign body is an occasional complication and was first described by Lambert in 1898.^3^ This diagnosis is challenging due to the nonspecific symptoms and imaging exams. A fatal outcome may ensue depending on the precocity of the diagnosis.^4^^,^ 5 The present report aims to demonstrate a case of a liver abscess, treated with a laparoscopic procedure and caused by the accidental ingestion of a twig.

## CASE REPORT

A 54-year-old male patient was referred to the emergency room with epigastric pain, dyspnea, fatigue over the last 13 days, and intermittent fever. His medical history included hypertension and dyslipidemia. At admission, he was in good general condition, afebrile, and tachycardic. The abdomen was tender on the right hypochondrium, but no signs of peritoneal irritation or visceromegaly were present. Acute cholecystitis was the initial diagnostic hypothesis.

The initial laboratory investigation showed leukocytosis with left shift, elevated inflammatory markers, increased hepatic enzymes, and negative serology (Table 1).

**Table 1:** Laboratory workup on admission

**Exam**	**Result**	**RR**	**Exam**	**Result**	**RR**
Hemoglobin	12.6	12.5-17.0 g/dL	AST	118	<35 U/L
Leukocytes	18200	3600-12000 /mm^3^	ALT	146	<45 U/L
Neutrophils	14560	1440-9000 /mm^3^	Total Bilirubin	0.76	0.3-1.2 mg/dL
Band	2002	0-600 /mm^3^	Direct Bilirubin	0.16	<0.2 mg/dL
Platelet	13x10^4^	15x10^4^0000-45x10^4^ /mm^3^	VDRL	NR	NR
CRP	180	<5 mg/L	Anti-HIV I and II	NR	NR
Creatinine	1.18	0.8-1.3 mg/dL	HbsAg	NR	NR
Urea	64.2	13-43 mg/dL	Anti-HBs	183	>10 mIU/L
Lactate	0.9	0.5-2.2 mmol/L	Anti-HCV	NR	NR

ALT=alanine aminotransferase; AST = aspartate aminotransferase; CRP = C-reactive protein; NR = non-reactive; RR = reference range.

**Figure 1:**
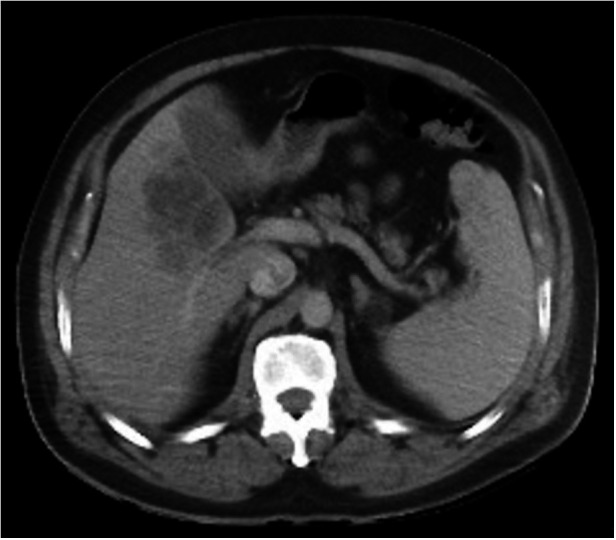
Abdominal computed tomography scan with intravenous contrast, portal phase, showing a hypodense and septated lesion in the hepatic segment V with peripheral enhancement.

The contrast-enhanced abdominal and pelvic computed tomography scan (CT scan) showed a hypodense and septated lesion in the hepatic segment V with peripheral enhancement, consistent with a liver abscess and with an estimated volume of 60 ml (Figure 1). He was hospitalized and promptly treated with intravenous saline, antibiotics (ceftriaxone + metronidazole) as the clinical status worsened.

On the second day, the clinical status improved. There was defervescence of fever and complete improvement of the abdominal discomfort, which was well evidenced in the initial five days of hospitalization. Abdominal ultrasound demonstrated a slightly thickened gallbladder wall with a 10 mm gallstone; meanwhile, it was not impacted in the infundibulum, generating doubt about the initial diagnostic hypothesis. Clinical and expectant treatment was maintained.

**Figure 2:**
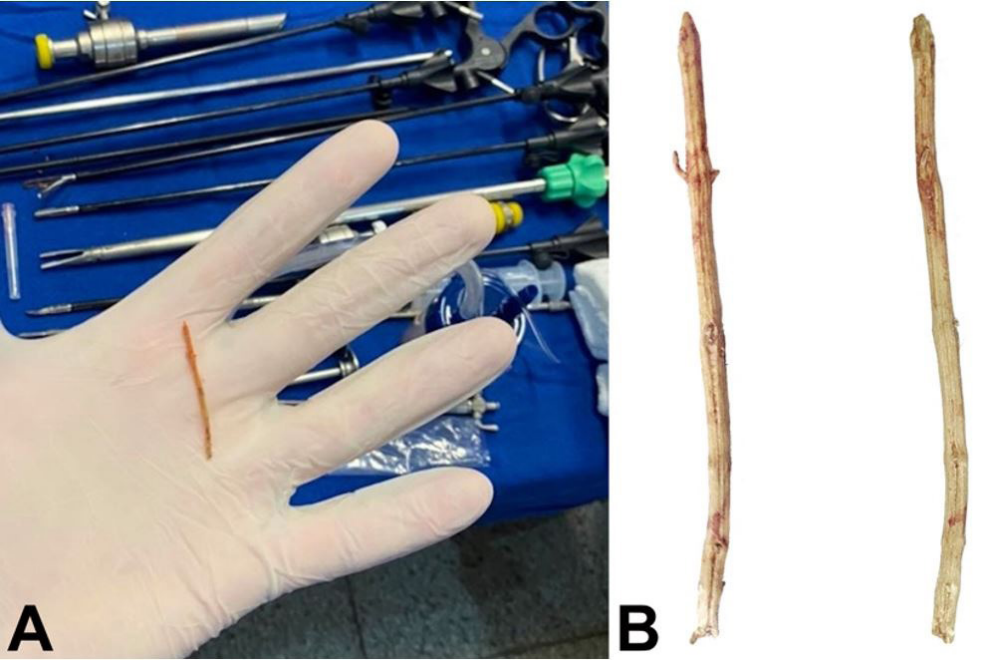
**A** –The intraoperative moment after laparoscopic removal of the foreign body; **B** – Frontal and lateral view of the foreign body (rosemary twig).

On day 6, a control CT scan showed an increase in the lesion volume (264 ml). Due to the unavailability to perform percutaneous drainage, the laparoscopic approach was performed. The procedure disclosed a thickened gallbladder wall, adhesions, and a hepatic bulging close to the gallbladder bed. After the release of adhesions, the abscess was drained – a thin and elongated foreign body of approximately 4.1 cm, resembling a rosemary twig (Figure 2), was found and removed from inside the liver abscess cavity (liver segment V). The abscess content was sent for culture. Despite not showing up complicated acute cholecystitis, because there was a cleavage plan between the gallbladder and the abscess, cholecystectomy was performed at the same surgical approach due to the reactive inflammation. No evidence of perforation of the gastrointestinal tract was observed. The abdominal cavity was drained by means of a tubulolaminar drainage.

The patient had a favorable outcome and was discharged on the second postoperative day with broad-spectrum oral antibiotic therapy (ciprofloxacin + metronidazole) for 21 days and an early outpatient appointment. The tubulolaminar drain was removed after ten days due to a small amount of secretion. The culture showed growth of gram-positive cocci, compatible with methicillin-sensitive *Staphylococcus aureus*; however, antimicrobial therapy was maintained because of the clinical improvement.

Two months after the surgical treatment, the liver abscess image could not be evidenced in the abdominal CT scan.

## DISCUSSION

Pyogenic liver abscess is characterized by a purulent intrahepatic collection of bacterial, fungal, or protozoan origin, which usually evolve in a subacute way and have an incidence of 1.1 to 2.3 per 100,000 inhabitants.^6^ Diabetes, cancer, cirrhosis, and immunosuppression are considered predisposing factors. The hepatic abscess is associated with diseases of the bile ducts, hematogenous dissemination through the portal vein or hepatic artery, local dissemination by other infected tissues, and traumatic injuries.^7^^,^ 8 The treatment must consider the cause, the service experience, and access to diagnosis and treatment devices. Antimicrobial therapy alone or associated with percutaneous drainage proved to be a safe and effective therapeutic resource for most hepatic abscesses.^6^

Cases of liver abscesses caused by foreign bodies are rare, with scarce reports in the literature. The foreign bodies mostly found in these abscesses are fish bones, chicken bones, and toothpicks.^8^^,^ 9 Hepatic abscesses associated with foreign body perforate mostly the stomach and the duodenum. The object is usually lodged in the left lobe of the liver due to anatomical proximity.^2^^,^ 4 The ascending and the transverse colon could also be perforation sites. However, in such cases, the object usually causes an abscess in the right liver lobe.^8^ In this report, no gastrointestinal perforation site or adhesions suggestive of perforation were observed; otherwise, as the abscess was found in the hepatic segment V, the antrum, the pylorus, the first and the second part of the duodenum, as well as the ascending and the transverse colon may have been the perforation site.

Hepatic abscess after foreign body ingestion is a challenging diagnosis. Most patients have nonspecific symptoms, and more than 90% of patients do not remember having ingested an object, which occurred accidentally.^1^^,^ 7 The most common symptoms are epigastric or abdominal pain, followed by fever. Chills, anorexia, fatigue, vomiting, nausea, and weight loss are also reported.^2^^,^ 8 Although ultrasonography and/or tomography are recommended for suspected diagnosis, hyperdense objects, such as fish bones, chicken bones, or metallic materials are more easily identified through CT scan; however, hypodense objects, such as wood materials, may not be visualized inside the abscess, as it occurred in the present report. Many reports have no foreign body detection through radiological images, so they are only diagnosed after surgery. Of these, exploratory laparotomy is the most cited modality.^2^^,^ 8 ^,^ 9

As the main treatment must involve the object removal, the hypothesis of a liver abscess caused by a foreign body should be questioned if there is a unique liver abscess refractory to antimicrobial therapy and/or percutaneous drainage.^8^^,^ 10

Regarding the identification of pathogens, Chong et al.^1^ reported a single bacterial strain in 54.5% of foreign body-related liver abscess cases. In their literature review, two or more pathogens were reported in 30.9%; *Streptococcus sp.* were the most commonly isolated pathogen (72.3%), followed by *Escherichia coli* (17%) and *Klebsiella pneumoniae* (10.6%). *Staphylococcus aureus,* the same pathogen of this case report, was found in 4.26% of the liver abscesses, and the probability of contamination must be considered. Even a rarer type, *Candida sp.*, was also reported.^1^

According to the Surgical Infection Society, the general principle of empirical antimicrobial regimen for intra-abdominal infection is activity against the typical gram-negative ﻿*Enterobacteriaceae*, gram-positive cocci, and anaerobes (Grade 1-A). Cefotaxime/ceftriaxone + metronidazole or ertapenem is recommended for empiric intravenous therapy in adults at low risk of microbial resistance (Grade 1-A). The substitution of intravenous to oral therapy should be performed with ﻿ciprofloxacin + metronidazole to complete a short course of antimicrobial treatment in these selective adults (Grade 1-B). Therefore, therapy modification based on culture results is not recommended in lower-risk patients who have had a satisfactory clinical response (Grade 1-B).^11^

In a systematic review about foreign body liver abscesses, Leggieri et al. 8 reported that most cases occur in men with a mean age of 54 years. Average size of objects at 4 cm, and leukocytosis with left shift and elevated C-reactive protein are the most commonly altered exams. Data are very similar to those found in the present case.

A Serbian case report presented by Karamarkovic et al. 12 showed a liver abscess in segment IVB-V caused by the ingestion of a 4.5 cm rosemary twig, diagnosed and treated by exploratory laparotomy. This report revealed a small perforation site at the gastric antrum wall, and the blood culture also showed *Staphylococcus aureus*. The foreign body found in this present report is compatible with the same origin. It was due to that Serbian article that the comparative investigation of the origin of the foreign body was carried out.

A literature review presented 22 cases of foreign body-related liver abscess treated by laparoscopy (Table 2). In all cases, the abscess was in the left liver lobe. Fishbones were the most found object (77.3%), followed by chicken bones (13.6%), toothpicks (4.5%), and sewing needles (4.5%). The most cited initial treatment was surgery because some patients were diagnosed with peritonitis, or the foreign body was seen on radiological images. Otherwise, many articles tried antibiotics or antibiotics + percutaneous drainage as the initial therapeutic choice due to the non-detection of the object in imaging exams. However, in all cases, the confirmed diagnosis was made after removing the object by the laparoscopic procedure.

**Table 2:** Literature review of laparoscopic treated foreign body liver abscesses

**Author**	**Country**	**Object**	**Liver lobe**	**Liver segment**	**Initial treatment**
Bekki^7^	Japan	Fish bone	Left lobe	III	Surgery
Abu-Wasel^9^	Canada	Toothpick	Left lobe	II and III	Antibiotics
B-de Mello^10^	Brazil	Fish bone	Left lobe	II and III	Antibiotics + PD
Beckers^13^	Belgium	Fish bone	Left lobe	IVA	Antibiotics + PD
Carver^14^	Canada	Sewing needle	Left lobe	N/A	Surgery
Rozanski^15^	Switzerland	Chicken bone	Left lobe	N/A	Surgery
Riani^16^	Belgium	Fish bone	Left lobe	N/A	Antibiotics + PD
Ricci^17^	Italy	Chicken bone	Left lobe	N/A	Antibiotics
Akimori^18^	Japan	Fish bone	Left lobe	N/A	Antibiotics
Koşar^19^	Turkey	Fish bone	Left lobe	N/A	Surgery
Panebianco^20^	Italy	Fish bone	Left lobe	N/A	Antibiotics
Morelli^21^	Italy	Fish bone	Left lobe	III	Antibiotics
Tan^22^	Singapore	Fish bone	Left lobe	II and III	Antibiotics + PD
Tan^22^	Singapore	Fish bone	Left lobe	N/A	Antibiotics + PD
Yu^23^	China	Fish bone	Left lobe	N/A	Antibiotics
Chen^24^	China	Fish bone	Left lobe	III	Surgery
Graça^25^	Portugal	Fish bone	Left lobe	N/A	Antibiotics
Barkai^26^	Israel	Fish bone	Left lobe	IVB	Surgery
Hernández^27^	Spain	Fish bone	Left lobe	N/A	Antibiotics
Rajaguru^28^	Singapore	Fish bone	Left lobe	IVB	Surgery
Cui^29^	Australia	Fish bone	Left lobe	IVB	Surgery
Hoff^30^	Australia	Chicken bone	Left lobe	I	Surgery
Index case	Brazil	Rosemary twig	Right lobe	V	Antibiotics

N/A= not available; PC= percutaneous drainage.

In summary, the recommended treatment for these situations is foreign body removal, associated with surgical drainage and antibiotic therapy. Although most of the reported surgical treatments are for laparotomy, some authors state that their choice is made for the laparoscopic approach because it is safer and less invasive.^7^^,^ 10 ^,^ 13 ^,^ 14

## CONCLUSION

Liver abscess caused by ingestion of a foreign body is a rare and challenging diagnosis; however, the diagnostic hypothesis should be considered in cases of refractory liver abscess. This report demonstrates a case of a liver abscess caused by a rosemary twig, properly diagnosed and successfully treated by means of laparoscopy.
